# Effects of a ketogenic diet on the quality of life in 16 patients with advanced cancer: A pilot trial

**DOI:** 10.1186/1743-7075-8-54

**Published:** 2011-07-27

**Authors:** Melanie Schmidt, Nadja Pfetzer, Micheal Schwab, Ingrid Strauss, Ulrike Kämmerer

**Affiliations:** 1 Dept. Obstetrics and Gynecology, University Hospital of Wuerzburg, Josef-Schneider-Str. 4, D-97080 Wuerzburg, Germany

**Keywords:** Ketogenic diet, cancer patients, pilot study, quality of life

## Abstract

**Background:**

Tumor patients exhibit an increased peripheral demand of fatty acids and protein. Contrarily, tumors utilize glucose as their main source of energy supply. Thus, a diet supplying the cancer patient with sufficient fat and protein for his demands while restricting the carbohydrates (CHO) tumors thrive on, could be a helpful strategy in improving the patients' situation. A ketogenic diet (KD) fulfills these requirements. Therefore, we performed a pilot study to investigate the feasibility of a KD and its influence on the quality of life of patients with advanced metastatic tumors.

**Methods:**

Sixteen patients with advanced metastatic tumors and no conventional therapeutic options participated in the study. The patients were instructed to follow a KD (less than 70 g CHO per day) with normal groceries and were provided with a supply of food additives to mix a protein/fat shake to simplify the 3-month intervention period. Quality of life [assessed by EORTC QLQ-C30 (version 2)], serum and general health parameters were determined at baseline, after every two weeks of follow-up, or after drop out. The effect of dietary change on metabolism was monitored daily by measuring urinary ketone bodies.

**Results:**

One patient did not tolerate the diet and dropped out within 3 days. Among those who tolerated the diet, two patients died early, one stopped after 2 weeks due to personal reasons, one felt unable to stick to the diet after 4 weeks, one stopped after 6 and two stopped after 7 and 8 weeks due to progress of the disease, one had to discontinue after 6 weeks to resume chemotherapy and five completed the 3 month intervention period. These five and the one who resumed chemotherapy after 6 weeks report an improved emotional functioning and less insomnia, while several other parameters of quality of life remained stable or worsened, reflecting their very advanced disease. Except for temporary constipation and fatigue, we found no severe adverse side effects, especially no changes in cholesterol or blood lipids.

**Conclusions:**

These pilot data suggest that a KD is suitable for even advanced cancer patients. It has no severe side effects and might improve aspects of quality of life and blood parameters in some patients with advanced metastatic tumors.

## Introduction

In the recent past, a remarkable growing interest could be observed in scientific literature concerning the striking carbohydrate metabolism of tumor tissue. In contrast to normal tissues, which can metabolize glucose, fatty acids and ketone bodies, according to Warburg's observations, many tumors depend heavily on glucose for their metabolic demands and ferment it to lactate - even under sufficient oxygen supply [1,2]. For this purpose, tumor cells have a remarkable up regulation of glucose transporter molecules on their surface. In addition, there is a frequent over expression of several key enzymes of glycolysis and attached pathways [3,4]. This prominent change in metabolism and associated enzymes/receptors could provide attractive targets for tumor-specific therapies. Several substances such as specific drugs interfering with the Warburg effect are under investigation [5-7]. However, to date, no safe and established therapy is available that targets tumor metabolism to fight cancer.

Hence, the search for alternatives to drugs is reasonable. Since 1885, when E. Freund observed that patients with malignant disease can develop spontaneous hyperglycaemia [8], there has been episodic interest in the association of the altered glucose metabolism with the path of nutrition and neoplasia in man. As early as 1924, Händel and Tadeuma summarized the findings in those days as: "a diet rich in carbohydrates has a pronounced stimulating impact on tumor growth" [9]. Reckoning the metabolic situation in the tumor patient's body, it could not be overseen, that increasing insulin resistance [10] and fatty acid oxidation [11] is characteristic for healthy tissue. In contrast, tumor cells often lack the ability to use fatty acids or ketone bodies (acetoacetate, beta-hydroxybutyrate) as an energy source and could even be harmed by them [12-15]. In an elegant in vivo setting, Holm et al have proven the different substrate utilization rates of tumor tissue and peripheral tissue in colon cancer patients. They clearly demonstrated that the peripheral tissue of the patients preferably utilized fatty acids and ketone bodies for energy demands, while the tumour showed the Warburg effect [16]. Thus, supporting fatty acid metabolism and inhibiting glucose metabolism should "feed" the body while neither supporting nor harming the tumor. LowCarb/HighFat (LCHF) diets and the strictly carbohydrate restricted ketogenic diet (KD) fulfill this purpose. Here, ketone bodies are produced as intermediate catabolic products of fatty acid breakdown by the liver.These ketone bodies can substitute glucose as an energy source in nearly every healthy tissue including the brain, even in a nutritional situation completely devoid of carbohydrates (CHO). This was impressively shown nearly one hundred years ago by the arctic explorer Vilhjalmur Stefansson, whose clinically controlled one year meat only diet impaired neither his nor his partner's physical and mental fitness [17].

Ketogenic diets have been used for the treatment of epilepsy in children since the 1920s [18]. Currently, the number of centers applying a KD to treat children with drug resistant epilepsy and the number of clinical studies have dramatically increased [19-21]. Other than classical KDs for the treatment of epilepsy which are in general 80% fat, 15% protein and 5% CHO, a KD with 60% fat, 30% protein and 10% carbohydrate was introduced by Atkins in the 1970s to combat obesity [22]. Nowadays, the Atkins diet is very popular and although it's long-term effect on weight loss is discussed controversially [23] undoubtedly it has a positive effect on the triglyceride and insulin levels [24,25] and no adverse side effects.

In 1995, two female pediatric patients with advanced stage astrocytoma tumors were treated at the Case Western Reserve University, Cleveland, Ohio with a KD based on medium chain triglycerides (MCT) as fat source [26]. The glucose uptake of the tumor decreased remarkably in both children and one of the patients was free of disease progression for 12 months of follow up and was still alive 10 years later (Nebeling L, personal communication). Several groups in the first decades of the last century reported that a diet low in carbohydrates and rich in fat and protein was an effective treatment in animal settings [9,27,28]. Although different in experimental details, all three groups agreed that withdrawal of carbohydrates and enrichment of fat in the chow fed ad libitum to tumor bearing animals led to a strong reduction in tumor growth. In this respect, data from the Seyfried lab demonstrated, that a calorie reduced KD was able to considerably reduce the intracerebral growth of malignant brain cancer cells in mice [13] and a tumor in a female glioblastoma patient [29], however, at the expense of a dramatic weight loss. Earlier, it was shown, that the ketone body beta-hydroxybutyrate not only inhibited the growth of several tumor cells in vitro, but also reduced the number of B16 melanoma deposits in the lungs of C57BL/6 mice by two thirds [30]. This inhibitory effect of beta-hydroxybutyrate and acetoacetate on tumor cell growth was confirmed in colon and breast cancer cell lines [14], as well as in neuroblastoma cells [15].

It has been shown recently, that a KD significantly decreased tumor volume and increased survival time in a mouse model for prostate cancer, compared to animals fed the standard "Western diet" [31]. Of importance, this effect was observed without restricting total calories and the mice did not lose body weight, a situation desirable in humans, especially in advanced cancer patients. In addition, a chow enriched in Omega-3 fatty acids, even if it was non-ketogenic, has been shown to reduce tumor growth rate and tumor cell proliferation significantly in animal models [32]. Our own preliminary experiments have shown that the application of an unrestricted ketogenic diet enriched with Omega-3 fatty acids and MCT delayed tumor growth in a mouse xenograft model [33].

Based on data from literature and our own observations, an LCHF diet was established to treat advanced cancer patients that restricted CHO to a maximum of 70 g/day, was enriched in fat - with emphasis on Omega-3 fatty acids - and nonrestricted in overall calories. The aims of the pilot study presented here were a) to prove the tolerability of such a diet in advanced tumor patients with no further established (classical) therapeutic options b) to see which effect it has on the quality of life, as determined by EORTC QLQ-C30 (version 2) [34] and c) to analyze the effects of such a diet on classical blood parameters and the course of disease. No specific tumor entity was chosen, the diet was offered to all patients who fulfilled the inclusion criteria.

## Material and methods

### Patients

The study was approved by the ethics committee of the Medical Faculty of the University of Wuerzburg, Germany. All participants signed an informed consent and obtained the approval of their home-oncologist.

Inclusion criteria for the study were:

1) advanced/metastatic tumor stage of solid malignant tumors of different origins,

2) no actual established therapeutic option available (no chemo- or radiotherapy),

3) measurable parameter for follow up (tumor-marker in serum or tumor visible in CT/PET/MRI),

4) acceptable general condition (as determined by the Karnofsky performance status (KPS) scale; [35])

5) laboratory values had to be within nearly normal range: red and white blood cell count, hemoglobin, platelet count, sodium, potassium, calcium, glycohemoglobin, AST (serum glutamic-oxaloacetic transaminase), ALT (serum glutamic-pyruvic transaminase), bilirubin, blood urea nitrogen, creatinine, uric acid, cholesterol, high and low density lipoproteins, triglycerides, lipase, total protein, albumin, prothrombin time, thyroid stimulating hormone. No signs of systemic inflammation (very high CRP and leukocyte counts) should be present.

Patients with mildly elevated (less than 20%) levels of liver enzymes, bilirubin, creatinine (up to 1.6 mg/dl), uric acid, blood urea nitrogen (up to 68 mg/dl), triglycerides, cholesterol or lipase were allowed in the study. Patients with slightly lowered (less than 20%) white blood cell count, hemoglobin, cholesterol or triglycerides were also allowed to participate. Low-dose thyroid stimulating hormone was allowed for patients with thyroid carcinoma.

6) Patients had to feel capable of following the dietary guidelines and shopping and cooking, perhaps with support from a family member.

The patients included in the study and their characteristics are listed in Table 1.

**Table 1:** Data of patients enrolled in the study

No	Age	Sex	Primary tumor	Measurement of disease	Metastases	Therapy between primary surgery and start of diet
1	47	f	Ovarian cancer	CT, CA 125	LI, LN, PC	10 × Taxol/Carboplatin; 10 × Hycamptin
2	46	f	Breast Cancer	PET	MPE, AS	Radiatio, 6 × [CMF]; 12 × [Epirubicin/Cyclophosphamid[, 14 × [Taxotere[, 2 × [Gemcetabine]
3	48	f	Granulosa cell tumor	CT, PET, Inhibin	LI, MI	6 × [Carboplatin/Epirubicin/Cyclophosphamid], 3 × Hemihepatectomy
4	30	f	Parotis carcinoma	CT	LO	Multiple surgery; Radiation; 6 × [Paclitaxel/Cisplatin]
5	62	f	Ovarian Cancer	US, CA 125	PC, FIGO IV	? × [Taxol/Carboplatin]
6	38	f	Osteosarcoma (jaw)	CT	LO	Multiple surgery
7	51	m	Oesophagus carcinoma	CT	LI, LN, MPE	2 × [Radiotherapy+Cisplatin/5-FU]; 2 × [Cisplatin/5-FU]; 7 × [Doxotaxel]
8	65	f	Pancreas carcinoma	MRI	LI	6 × [Gemcetabine], Immunotherapy (Survivin)
9	33	m	Thyroid carcinoma	US, CT, Calcitonin	LI, BO	Sanostatin, Interferon
10	50	m	Pancreas carcinoma	PET	LI	CapRI-Study branch A (Radiotherapy, Cisplatin/5-FU, IFN-alpha)
11	64	f	Thyroid carcinoma	CT, TG	LU, LN	Radio-Jod Therapy, Sanostatin
12	42	f	Colon carcinoma	PET	LI, LU	6 × Radiotherapie (38,6 GBq I-131); Avastin; ? × [Cisplatin/Carboplatin]
13	54	f	Endometrial cancer	CT	LI, PC, AS	8 × [Cisplatin/Adriamycin]; 2 × [Adriamycin/Doxorubicin]; 6 × [Navelbine/Carboplatin]
14	60	f	Lung cancer	PET	LI	6 × [Carboplatin/Cisplatin/Etoposid]
15	62	m	Stomach cancer	PET	LI, PE, AS	1 × [Irinothekan/5-FU/Folinacid]; 5 × [Etoposid/5-FU/Folinacid]
16	54	f	Ovarian cancer	CA 125	PC, AS, Figo IIIC	? × [Taxol/Carboplatin]

LU: lung metastases, LI: liver metastases, LN: lymph node metastases, BM: bone metastases; MI: metastasis in Mediastinum; PC: peritoneal cancerosis, AS: ascites, MPE: malign pleural effusion; LO: local progress; × [...]: cycles of chemotherapy

### Study design

**Figure 1:**
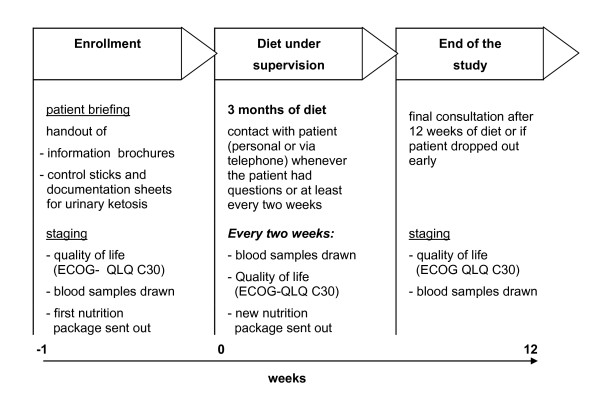
**Study design**. The course of the study plan, including the intervals of data acquisition is shown in this diagram.

Type of study: Prospective observational pilot study to investigate if a diet very low in CHO and rich in fatty acids is safe and feasible for advanced cancer patients; without adverse side effects and with improvement of their quality of life. The course of the study is shown in Figure 1.

### Nutrition

The patients started the diet after having been thoroughly instructed in the principles and practical realization of the diet. A dietary manual containing background information, a table with nutritional contents of the most important foods, a food exchange list and recipes for baked goods containing extremely low amounts of CHO was provided to the patients to assist with education and compliance. For variety in the daily menu, a regularly up-dated brochure containing recipes for cakes and sweets consistent with the diet's principles was provided. In general, the amount and type of food intake was permitted ad libitum, however, with several essential guidelines:

a) The overall CHO intake was limited to 70 g per day and 20 g per meal (the patients were instructed to reduce their CHO even more, if possible). In order to fulfill this precept, the patients were briefed to follow the essential rules given in Table 2.

**Table 2:** Dietary guidelines for the patients

Rule	Description
1	Avoid all types of bread, cake, processed snacks, sweets, potatoes, pasta, rice, polenta, vegetables rich in starch (corn, beans, peas) and cereals.
2	Be aware of hidden sources of CHO in sugar sweetened drinks, candy, chewing gum with sugar, milk and milk products, lunch meat and some cheeses as well as in most "low fat" products.
3	Fruits are rich in CHO, therefore always calculate the amount and select those which are low in CHO.
4	Vegetables are often rich in CHO - but mainly in dietary fiber, therefore calculate the usable CHO only.
5	If possible, prefer cold-water fish and meat from grazing cattle as protein sources, because of their preferable fatty acid pattern
6	Vegetables and the few fruits allowed should be grown organic
7	As nibbles, select oil-rich nuts (walnuts, brazil nuts, macadamia nuts) and seeds (sunflower), and only occasionally chocolate with very high cacao content (min. 85%).

b) The diet plan contained two substantial liquid meals to be taken as snacks morning and afternoon. These meals were an oil-protein shake with three components: 250 ml highly fermented yoghurt-drink, 8 ml vegetable oil-mixture and 10 g protein preparation (all Evomed, Darmstadt, Germany, Table 3). The components were provided via a nutritional package given to the patients every two weeks and stored at 4-8°C. The oil-protein shake was prepared freshly in a blender by the patient and provided 264 kcal gross energy with 21 g of fat (including 1.3 g omega-3 fatty acids), 5 g of CHO and 14 g of protein and was well tolerated by all patients. In order to combat cachexia [36] and to add a maximum of Omega-3 fatty acids, patients were encouraged to add additional servings (1 tablespoon each) of the oil mixture or other oils from olives, flaxseed and hempseed to the three principle meals, e.g. added to salad or curd cheese.

**Table 3:** Composition of the additive foods supplied during the study

	Highly fermented yoghurt-drink	Vegetable oil mixture	Protein preparation
Ingredients	skimmed milk	line seed oil	milk-protein
	plant oil mixture	canola oil	
	pectin	walnut oil	
		MCT	
		grape seed oil	
		argan-oil	
		pumpkin seed oil,	
Energy per 100 g	245 kJ/59 kcal	3730 kJ/891 kcal	1550 kJ/370 kcal
Protein	1,5 g	0	88-90,3 g
Fat	5,1 g	99,9 g	1 g
Saturated FA	1,3 g	36,5 g	n.a.
Unsaturated FA	3,8 g	63,4	n.a.
Omega-3 FA	0,3 g	19,6 g	n.a.
CHO	1,7 g	0	0,2 g

n. a. = not analyzed; FA: fatty acids, CHO: carbohydrates, MCT: medium chain triglyceride.

### Evaluation

The patients were staged by their attending oncologist before and at the end of the study to monitor the course of the disease. The method of staging depended on the primary tumor and the known metastatic sites. For some patients we were able to monitor the course of the disease via tumor markers as well. Information on staging parameters and tumor markers can be found in Table 1. The effect of the LCHF diet on ketone body production was measured by the patients themselves (early morning self assessment of ketonuria; Keto Diastix; Bayer Health Care, Leverkusen, Germany) and documented in a special table handed out at their first briefing. A stable ketonuria was when at least 0.5 mmol/l of ketone bodies were documented on more than half of the days.

Lipid levels, electrolytes, kidney and liver functions and hematologic parameters as described in the inclusion criteria were assessed at baseline and every two weeks, or at time of drop out.

To assess how the disease affects the daily life of the patient, the performance status was monitored by the EORTC QLQ-C30 (version 2) questionnaire [34] developed by the European Organization for Research and Treatment of Cancer [37]. The EORTC QLQ-C30 is designed to evaluate the global health status, with functional scales including physical, role, emotional, cognitive and social functioning as well as symptom scales including fatigue, nausea and vomiting, pain, dyspnoea, insomnia, appetite loss, constipation, diarrhoea and financial difficulties. The questionnaire was completed by the patients at the beginning of the study, after every two weeks, and at the end of the study. We evaluated quality of life for all patients who completed the EORTC QLQ-C30 after at least two months on the diet.

### Scoring and Statistics

The EORTC QLQ-C30 questionnaires were scored as described in the EORTC QLQ-C30 Scoring Manual [38]. Blood parameters and body weight were analysed for significance using the two-tailed student's t-test and the Prism 4.0 software (GraphPad; Statcon, Witzenhausen, Germany) before and after the diet. Being aware of the explorative nature of the data obtained, probability values below 0.02 (global alpha p = 0.05 corrected following the Bonferroni-Holm process for multiple test procedures [39]) were considered significant.

## Results

### Trial profile

**Figure 2:**
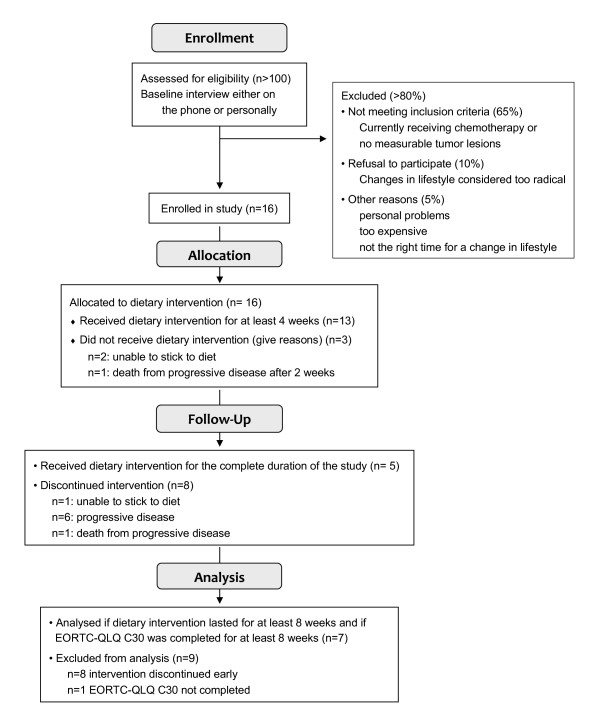
**Trial profile flow diagram**. A trial profile corresponding to the CONSORT criteria is shown indicating the course of the pilot study

A trial profile according to the CONSORT-statement (http://www.consort-statement.org) criteria is given in Figure 2, althought this study was not a randomized, controlled trial.

### Recruitment of patients

Announcing the study provoked a barrage of applications from interested cancer patients. However, the majority of the patients interested were still under standard therapy or even in a disease free state and thus, due to the strict inclusion criteria, participation could be offered to very few of them. Of those, only 16 felt capable of following the dietary guidelines and thus, were enrolled in the study. The mean age of patients was 50.4 years (30-65 years). Among them were 12 women and 4 men with tumors of different entities as listed in Table 1. All patients enrolled were tumor patients with advanced to very advanced disease and they had already completed several therapies (operations, chemo- and radiotherapies). No further conventional therapeutic options were available to them at inclusion time. The patients were informed and agreed to participate in the study.

### Course of the diet

As observed in previous trials with ketogenic diets in adults [40], some patients found it very difficult to comply with a treatment that demands major changes in lifestyle. A problem mentioned frequently was the incorporation of the diet in the context of family life. The course of the patients' diet, including reasons for quitting, is summarized in Table 4. Five (31%) of the patients concluded the study per protocol. The other patients dropped out of the study earlier. Two of the patients withdrew from the study during the first two weeks because they were unable to adhere to the diet (patient 1) or because of personal problems (patient 2). Most of our patients were end stage tumor patients and two patients died from their malignant disease during the study (patients 7 and 8). One patient dropped out because he suffered from excessive weight loss and weakness (patient 10). One patient dropped out of the study early feeling no longer able to stick to the dietary guidelines (patient 14), one because of resuming chemotherapy (patient 7) and four abandoned the diet due to progress of their advanced cancer situation (patients 4, 6, 13 and 15).

**Table 4:** Duration of study, reasons for drop out

No	Duration of diet (weeks)	Ketosis > 0.5 mmol/l (% of days)	Diet rating	EORTC > 2 months	Laboratory parameters evaluated for statistics	Result	Reason for drop out
1	< 1	-	-	-		?	Drop out after 3 days because of vomiting, fatigue
2	< 2	-	+	-		?	Drop out after 10 days because of family problems
3	**12**	**61%**	+++	yes	yes	**SD**	
4	8	-	+	yes	yes	progress	Impaired food intake
5	**12**	25%	++	yes	yes	**SD**	
6	6	**97%**	++	-	yes	progress	Impaired food intake
7	2	-	o	-	-	death	
8	5	-	-	-	-	death	
9	**12**	**78%**	++	yes	yes	**SD**	
10	6	22%	+	-	yes	progress	Very advanced stage with fatigue and eating problems
11	**12**	25%	++	yes	yes	**SD**	
12	7	44%	++	-	yes	progress	Resumption of chemotherapy
13	8	**88%**	++	yes	yes	progress	Massive ascites, impaired food intake
14	4	-	o	-	-	?	Felt unable to continue the diet
15	7	**60%**	++	-	yes	progress	Impaired food intake
16	**12**	**100%**	+++	yes	yes	**SD**	

SD: stable disease; diet rating: [+++] very good; [++] good; [+] moderate; [-] poor/not feasible; [o] no comment on feasibility

**Figure 3:**
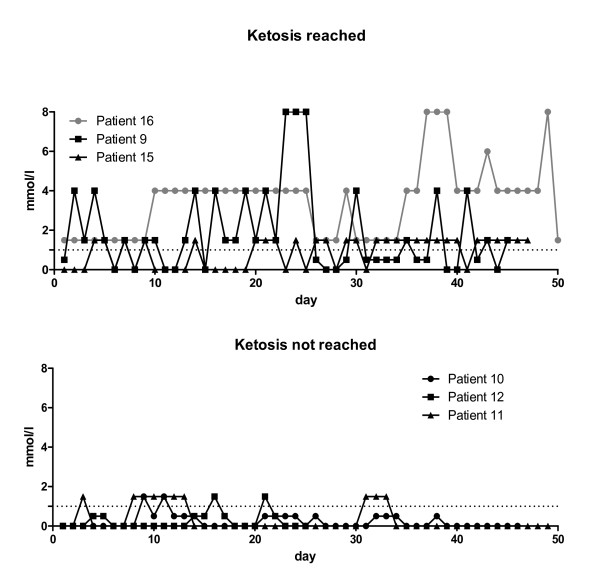
**Course of urinary ketones**. Typical examples of urinary ketone data are shown for three patients reaching ketonuria (A) and three who failed to reach ketonuria on the majority of days (B). The first 50 days of the intervention period are shown

All five patients who completed the study were in a stable disease, however, one of them failed to document the EORTC QLQ-C30 questionnaires regularly. Of those keeping to the diet for the whole 12 weeks, 3 (60%) reached a stable ketonuria as measured by early morning self assessment. Ketonuria was found to vary between 0.5 and 8 mM/l, predominantly being 1.5-4.0 mM. Stable ketonuria was evident in three other patients who, however, dropped out due to progression after 6-8 weeks. Characteristics of typical ketone measurements are shown for three patients reaching ketosis (Figure 3A) and three patients failing to reach ketosis (Figure 3B) during the first 50 days of diet.

Feedback from patients showed some problems with the realization of the diet. Moreover, the acceptance of the diet varied greatly. We asked the patients how they rated the feasibility of the diet after at least two weeks of dieting: very good, good, moderate, poor or very poor. One patient stated after three days that it was not feasible at all and stopped the diet. Two patients did not comment on feasibility, two patients rated it very good, 7 patients rated it good, three moderate and one poor.

### Quality of life

**Figure 4:**
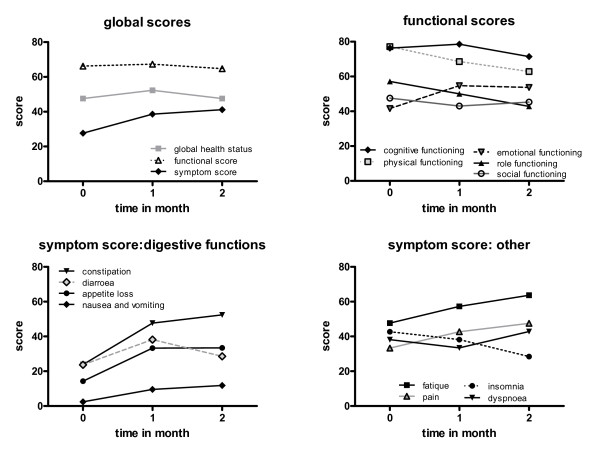
**Quality of life**. Summary of the EORTC QLQ-C30 (version 2) questionnaires. A): Global health status and functional score remained relatively stable during the time evaluated while the global symptom score increased slightly. B) Subdivision of functional scores shows a slight decrease in physical and role functioning, an increase in emotional functioning and stable cognitive as well as social functioning. C) Concerning digestive functions, the symptoms score showed an increase in appetite loss, constipation or diarrhoea in the first four weeks of the diet. Symptoms then remain stable and signs of diarrhoea diminish. D) The other symptoms were an increase in fatigue and pain corresponding to the very advanced tumor situation. However, insomnia clearly decreased with ongoing dieting. Graphs are given as means for the seven evaluable patients.

The initial quality of life, as measured by the EORTC QLQ-C30 questionnaire, was low in our patients due to their advanced tumor stage. The global scores, however, remained relatively stable throughout the evaluation time (Figure 4A). In addition, physical and role functioning worsened only slightly over time (Figure 4B). In patients with normal bowel function, the diet caused constipation (Figure 4C), whereas those patients suffering from diarrhoea as a symptom of their malignant disease observed a change for the better on the diet. Five out of 16 patients suffered from progressive disease and two patients died during the study. Thus, it was not surprising that the symptoms such as fatigue, pain or dyspnoea increased over time (Figure 4D). However, it was striking that emotional functioning increased slightly and insomnia improved (Figure 4B+4D). Therefore, at least some of the patients felt better on the diet.

### Blood parameters and leukocyte count

**Figure 5:**
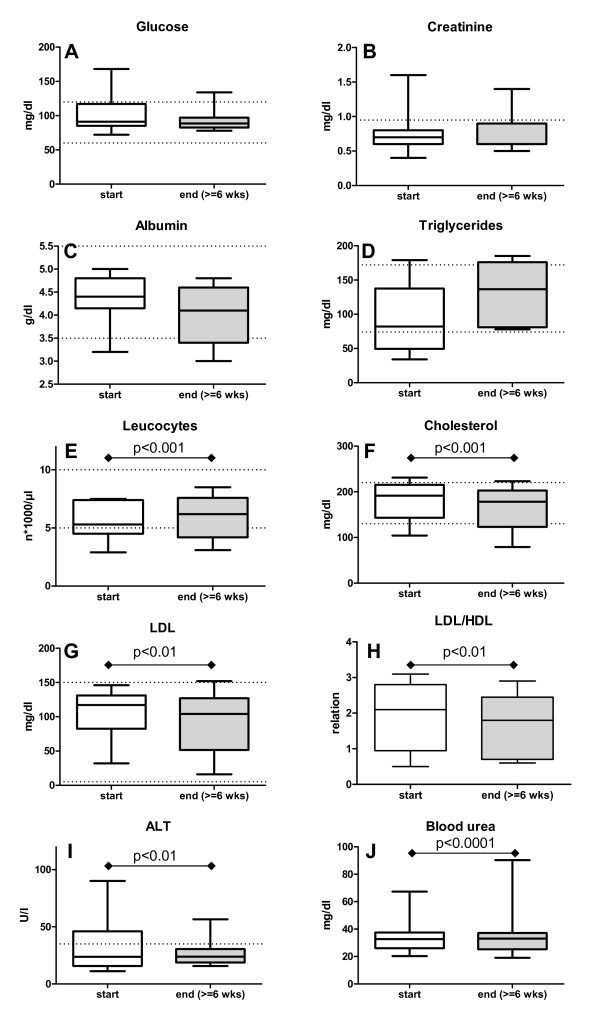
**Blood parameters**. The courses of the blood parameters for the 11 evaluable patients at start of study and after at least 6 weeks of dieting are shown. While the blood glucose (A), creatinine (B), albumin (C) and triglycerides (D) did not change much, leukocyte count increased significantly (E) and cholesterol (F), LDL (G) and LDL/HDL (H) decreased significantly. The patients', ALT dropped significantly (I) while blood urea increased, albeit remaining in normal range (J). Dotted lines: upper and lower level of normal range.

The laboratory parameters at enrollment and after the intervention period are given in Table 5. CRP was analyzed at time of enrollment, and two patients had mildly elevated CRP (patient 4: 8 mg/dl and patient 8: 4 mg/dl), while for the majority only a slight increase over normal range could be observed (0.9 mg/dl ± 0.7 SD). For those patients sticking to the diet for at least 6 weeks, data were available for six of the patients analysed for the EORTC QLQ-C30 questionnaires as well as for five additional patients, all of which were analyzed for significant changes. Data are shown in Figure 5.

**Table 5:** Blood parameters

Param.:		CRP	Glucose		HBA1c		Chol.		LDL		HDL		Triglyc.		Crea.		Urea		ALT		Alb		Leuc.	
**Units**		**mg/dl**	**mg/dl**		**%**		**mg/dl**		**mg/dl**		**mg/dl**		**mg/dl**		**mg/dl**		**mg/dl**		**U/l**		**d/dl**		N*1000/µl	
**Norm-range**		**0-0, 5**	**<120**				**130-220**		**0-150**		**> = 35**		**74-172**		**0 - 0,95**		**10 - 50**		**< = 35**		**3,5-5,5**		**5 - 10**	

**No**	**wk**	**S**	**S**	**E**	**S**	**E**	**S**	**E**	**S**	**E**	**S**	**E**	**S**	**E**	**S**	**E**	**S**	**E**	**S**	**E**	**S**	**E**	**S**	**E**

1	< 1	0,77	109	-	-	-	-	-	-	-	-	-	-	-	0,6	-	28,6	-	50,4	-	4,3	-	7,0	-
2	< 2	0,6	86	-	-	-	231	-	130	-	88	-	63	-	0,7	-	32,1	-	16,2	-	4,2	-	3,6	-
3	**12**	0,6	91	90	5,8	5,3	104	119	32	38	65	65	34	78	0,8	0,7	36,4	42,6	23,1	25,3	4,4	4,1	4,5	4,2
4	8	8,1	117	103	-	5,7	-	201	-	129	47	49	88	80	0,4	0,5	23,0	19,0	24,0	29,0	-	-	5,4	6,3
5	**12**	0,4	91	87	5,6	5,4	197	204	115	104	46	52	178	-	0,7	0,6	27,3	23,0	29,0	19,0	4,4	4,8	7,5	6,2
6	6	-	72	-	5,4	-	139	-	58	-	74	-	34	-	0,6	0,6	20,3	29,1	15,5	17,8	5,0	4,8	5,5	7,6
7	2	1,91	84	-	5,7	-		-	-	-	-	-	-	-	0,7	-	12,1	-	10,3	-	3,4	-	4,2	-
8	5	4,0	101	89	-	-	-	-	-	-	-	-	-	-	0,7	0,7	26,9	49	12,4	16,0	4,0	3,6	7,3	11
9	**12**	1,9	87	78	4,7	4,9	-	79	-	16	-	26	-	185	0,7	0,8	27,0	31,2	58,0	56,6	4,8	4,0	2,9	3,1
10	6	-	168	79	5,3	-	190	193	124	117	52	40	71	179	0,8	1	40,9	35,4	90,2	-	4,2	-	7,4	8,5
11	**12**	0,18	112	94	5,4	-	221	178	117	81	85	80	97	84	0,8	0,6	30	26	15,7	15,7	4,3	4,4	5,2	5,8
12	7	-	94	95	4,7	-	-	-	146	152	-	-	-	-	0,6	0,6	-	-	46	29	-	3,0	4,1	3,1
13	8	0,89	159	84	5,5	-	193	165	117	-	40	-	179	159	1,6	0,9	35,4	35,0	11,2	19,3	3,2	3,2	7,4	8,5
14	4	-	106	-	-	-	-	-	-	-	-	-	-	-	-	-	-	-	-	-	-	-	-	-
15	7	0,1	85	134	5,2	-	155	127	107	65	35	39	65	114	1,4	1,4	67,4	90,3	23,8	35,2	4,1	3,6	5,3	6,0
16	**12**	2	79	85	5,5	-	231	223	138	125	77	65	82	167	0,7	0,6	35,7	35,0	22,8	22,4	4,8	4,1	5,1	6,5
**n**		**12**	**16**	**11**	**11**	**4**	**9**	**9**	**10**	**9**	**10**	**8**	**10**	**8**	**15**	**12**	**14**	**11**	**15**	**11**	**13**	**10**	**15**	**12**
**ME**		**1,8**	**103**	**93**	**5,3**	**5,3**	**185**	**165**	**108**	**92**	**61**	**52**	**89**	**131**	**0,8**	**0,8**	**31,7**	**37,8**	**29,9**	**25,9**	**4,2**	**4,0**	**5,5**	**6,4**
**SD**		**2,3**	**27**	**16**	**0,4**	**0,3**	**44**	**48**	**36**	**45**	**19**	**17**	**51**	**47**	**0,3**	**0,3**	**12,6**	**19,4**	**22,2**	**11,9**	**0,5**	**0,6**	**1,5**	**1,2**

No: patient number; wk: weeks of diet; S: start of study; E: end of study, first blood at > = 4 weeks; ME: mean; SD: standard deviation

Two patients had elevated glucose levels before starting the diet, that returned to normal (patient 10 from 168 to 79 mg/dl; patient 13: 159 to 84 mg/dl). Two had slightly elevated cholesterol levels before the diet (patient 11: 221 mg/dl, patient 16: 231 mg/dl), which dropped during the diet (patient 11: 178 mg/dl, patient 16: 223 mg/dl), one had slightly elevated blood triglycerides which normalized through the course of the diet (patient 13 from 179 to 159 mg/dl) and one had elevated liver parameters (serum glutamic-pyruvic transaminase, ALT; patient 11) which fell during the intervention period from 46 U/l to within normal range, 29 U/l. Of the two patients exhibiting excessively low leukocyte counts, one (patient 9) showed a slight increase while one (patient 12) showed a further reduction in this parameter.

Analyzing all patients, overall blood cholesterol and HDL as well as LDL levels were reduced significantly after 6 weeks of dieting (see Table 5 and Figure 5). Since the reduction of LDL (108 ± 36 SD to 92 ± 45 SD mg/dl; p < 0.01) was more pronounced than that of HDL (61 ± 19 SD to 52 ± 17 SD mg/dl; p = 0.02), the LDL/HDL relation as a cardiovascular risk indicator decreased in the patients (from a mean of 1.9 ± 1.0 SD to 1.7 ± 0.9 SD; p < 0.01). The serum triglycerides increased from 89 ± 51 SD in mean to 131 ± 47 SD in mean, albeit the difference was not significant due to the broad variance of data. While the level of creatinine and albumin in sera remained stable, the blood urea nitrogen increased from 31.7 ± 12.6 SD to 37.8 ± 19.4 SD mg/dl (p < 0.0001), still within normal range. ALT as parameter for liver function declined to the better from 29.9 ± 22.2 SD to 25.9 ± 11.9 SD (p < 0.01). The total leukocyte count significantly increased from 5.5 ± 1.5 SD to 6.4 ± 1.2 SD × 1000/µl; p < 0.001 during the observation time.

One patient (13) suffered from diabetes as concomitant disease and reported a decreased (75% of initial units) insulin requirement during the study. With the exception of one patient (15), all patients showed reduced blood glucose levels on the diet.

### Body weight, BMI, albumin

The body weight of 7 patients was obtained at starting point and after 6-8 weeks of dieting. As described for diets low in CHO, all patients significantly lost weight during the diet. However, with the exception of one patient (no. 13) who intended to use the diet to reduce her obesity and lost 10 kg (in part, the extreme weight fluctuation observed in this patient was generated by recurrent ascites which was punctured three times during the study), the patients lost an average of 2 kg (from 68.5 ± 6.8 SD to 66.5 ± 6.8 SD; p < 0.05); consequently BMI was reduced from mean 23.5 ± 6.0 SD to 22.5 ± 5.4 SD.

### Influence on the tumor itself

A statistical evaluation of the effect of the diet on tumor characteristics is not feasible, due to the low number and heterogeneity of patients enrolled in our study. Instead, a description of the course of disease is given: Four patients who dropped out of the study early were not evaluated, two patients died early. Progress of disease occurred in 5 patients who then discontinued the diet, whereas 5 of the patients who adhered to the diet throughout the study had stable disease (Table 4).

## Discussion

In this pilot study, we evaluated the safety (occurrence of side effects), feasibility (following the dietary guidelines for advanced cancer patients) and impact (on body weight, laboratory parameters, quality of life) of a LowCarb/HighFat (LCHF) diet very low in carbohydrates (CHO) and rich in fat and protein on a heterogeneous group of advanced cancer patients. Announcing the study provoked a broad interest of media and the hospital was overwhelmed with enquiries from cancer patients. This reflects the enormous interest of cancer patients in complementary and alternative methods of improving their situation via change of lifestyle and, especially, their diet [41,42]. However, most of the patients interested were either end-stage (hoping for a "miraculous cancer cure") or disease free after primary therapy, intending to use the diet for prevention of relapse. Both groups were not eligible, thus reducing the number of patients fulfilling the requirements to very few. It should be noted in this respect, that due to the heterogeneity of the patients included and the nature of this diet with normal food, this study was a series of cases and neither randomized nor blinded.

As described in previous studies [40], we found an LCHF treatment in adult patients to be only slightly feasible. Based on our observations, an LCHF diet is not an option for all patients with advanced cancer, since the associated changes in eating habits (e.g. waiving soft drinks and beer) are not acceptable for some of them. For other patients however, an LCHF diet might be an option for increasing their quality of life. The latter belong to the group of patients who want to actively influence the course of the disease (by change of lifestyle) and who are in a phase of their disease where cooking and eating are not hampered. Although there was a worsening in some parameters of the quality of life assessment, reflecting the very advanced situation of our patients, we found an improvement in emotional functioning and insomnia, even though the course of the disease in our patients was progressive or in the best case, stable. Certainly, we cannot exclude the possibility of a placebo effect caused by a) the intensive consultation and briefing of the patients and b) the chance for patients to actively participate in their therapy. Further, anecdotic evidence links the presence of ketone bodies to a mild euphoria [43] which was assumed to be caused by one of the ketone bodies, beta-hydroxybutyrate. Thus, this metabolic state of ketosis, which was reached by six of our patients, could be the reason for the improvement in our patients, too.

Diets high in fat (up to 90%) and very low in CHO, inducing a stable ketosis, have been used for a long time to treat epilepsy and adiposity. The most frequent side effects reported so far are constipation, vomiting, lack of energy and hunger [20]. These symptoms were reported by our patients slightly more often within the first 4 weeks of the diet. However, none of them complained of hunger, and nausea and vomiting were very rare. We could also not determine from our data, whether the observed increase in fatigue was caused by the diet or by the progress of disease. In this context we can also report, that patients outside the study and following the diet during standard therapies (chemo-, radiotherapy), regularly report an increase in energy and condition (not shown).

The time frame of the study was selected according to common duration of studies dealing with the application of ketogenic diets in epilepsy patients [20,40] and based on the short survival perspective of the patients enrolled. Further, the Nebeling study showed that the effects of a ketogenic diet were not visible until after 8 weeks [26]. Since our patient group was small and diverse, we cannot comment on the influence of the LCHF diet on the course of disease. The influence of the diet on specific tumor entities or on tumors with different molecular characteristics concerning glucose metabolism must be evaluated in further studies.

The present study has several limitations. Only few patients fulfilled the requirements and all of them were in a very advanced stage of disease, as reflected by the two early death cases and the progress of disease in five cases which made it impossible for those patients to follow the diet for the whole time. Since we aimed to test the acceptability and compatibility of an LCHF diet in advanced cancer patients, we did not select patients according to their tumors. Therefore, the group was very inhomogeneous. Further, the majority of the study participants were not from our own hospital, but scattered all over Germany and blood samples and laboratory parameters had to be provided by their family doctors or local oncologists.

Compared to KD regimens for the treatment of epilepsy or obesity that reduce the amount of CHO allowed to at least 10-25 g per day, our LCHF protocol allowed up to 70 g CHO/day. This larger amount was allowed due to several considerations: 1) The daily glucose production rate in healthy patients was determined to be around 3.6 µmol/kg/min (corresponding to 0.933 g/kg/day) with a very stable rate of gluconeogenesis of 2.6 µmol/kg/min (corresponding to 0.67 g/kg/day) and a variable rate of glycogenolysis. The latter depends on the amount of glucose/protein and, if too much CHO was taken up, glucose was stored as glycogen [44]. 2) Increased rates of gluconeogenesis, which burns the body's lean mass and harms the patient, have been documented in patients with malignant disease [45]. Thus, advanced cancer patients hypothetically may tolerate a little more CHO in their diet without leaving the metabolic state ketosis. 3) The larger amounts of CHO allow patients to add yoghurt and some vegetables/fruits, which although containing milk or fruit sugar, are also promoted to be beneficial to cancer patients [46] and - based on the popularity of these books - very welcomed by our patients. These facts in mind, allowing a little more CHO in the diet should not severely influence the ketogenic effect and indeed, 6 of the 11 patients on the diet for at least 6 weeks reached ketosis. More CHO facilitates the selection and compilation of food and thus increases the compliance of the patients. From data available, we cannot determine the reasons for the failure of the remaining patients to reach a stable ketosis. It could be speculated in this respect, that either the amount of CHO allowed was over the individual ketogenetic limit, or the patients consumed additional CHO without documenting it. The method for analyzing urinary ketones does not seem to be the cause of negative results, since this method shows values that correlate highly with blood ketone values in a preliminary test series (not shown) and was already described in literature to show a good correlation to blood ketones in long time ketosis [47]. However, to help achieve a stable ketosis state in further trials, a step-by step induction of ketosis as suggested by Atkins [22] may be necessary, with a CHO content of the diet customized to the preservation of ketosis in each individual patient.

The significant reduction of body weight (and BMI) observed in all patients could reflect the typical effect observed in the early phase of low-carbohydrate diets - usually not restricted in calories. Here, the maximum weight loss was normally observed within the first 6 months of the diet [25,48,49]. In contrast to the weight loss observed in our group and described in general for ketogenic diets, a non-ketogenic high-fat diet (mixture of normal meals with an additional fat-enriched artificial liquid diet) supported the maintenance of body weight in patients with gastrointestinal tumors [36]. However, those patients were not "end stage" and the diet was followed during chemotherapy. Yet, based on their findings, it could be speculated that the application of an LCHF diet in an earlier phase of tumor disease might be of greater benefit for the patient.

Among the study patients and treating oncologists, many concerns were expressed that a diet very rich in total fat could adversely affect blood lipid levels or immune status. In accordance to data published in several extended studies applying low-carb diets to obese patients to lose weight [25,48,49], the cholesterol and especially LDL/HDL values of our patients significantly improved. The exception to this pattern is the triglyceride concentration. Other than described in the cases of ketogenic diets for the reduction of body weight [49], the levels of triglycerides in our patient's sera increased on the diet, albeit not to a significant level, and still in normal range. This effect was also observed by Mosek [40] and thus may reflect a normalization of the triglyceride level in non-overweight patients. However, we have not investigated subpopulations of immune cells, other than described in Breitkreutz [36] who observed a significant decrease in total leucocyte count in the group of patients on the fat rich diet. The leucocyte count in our patients significantly increased to the better. This difference may be due to different settings and the accompanying chemotherapy of the patients in the former study.

Since several studies have shown that supplementation with Omega-3 fatty acids (FA) benefits patients with advanced cancer and weight loss [50-52], the vegetable oil used in the oil-protein shake taken by the patients two times a day was especially enriched in Omega-3 FA. Further, the patients were encouraged to snack on nuts and seeds rich in Omega-3 FA such as flaxseed, hempseed and walnuts. However, the study was too short and the patients presumably too advanced in their disease to observe a beneficial effect on tumor growth as described in animal models [31,32,53].

When we started the study in 2007, except for two preliminary reports [26,54], no protocol was available on how to perform an LCHF or ketogenic diet study with cancer patients. Since then, a study protocol was published by Fine et al. [55], and four clinical trials were registered in the clinical trials database [56]. However, no patient's data resulting from these studies have been published so far. Thus, it will be very interesting to see if the slightly different nutritional settings of LCHF or ketogenic diets will benefit the study patients as well.

## Conclusions

A carbohydrate-restricted fat rich diet was well tolerated by 5 of 16 patients for 3 months and another 7 for at least 5 weeks until progression or death. No adverse laboratory effects were observed, but there was ongoing weight loss. The data of this pilot study further suggest that a KD might improve quality of life and classical blood parameters in some patients with advanced metastatic tumors. However, to judge effects on quality of life or cancer progression, randomized studies with sufficient numbers of patients are needed.

## Competing interests

All authors declare that there is no conflict of interest. This study was supported by the Medical Faculty of the University of Wuerzburg and by a grant of the charity organisation "Hilfe im Kampf gegen Krebs e. V.", Wuerzburg. The Evomed-Company (Darmstadt, Germany) contributed the nutritional packages for the patients, but neither the hospital nor any of the authors has any relationship with this company.

## Authors' contributions

MeS headed the study, enrolled and advised the patients and drafted the manuscript, NP assisted in advising the patients, supported the patients with nutritional day plans and cooking recipes and assisted in data analysis, MiS analyzed the patients data, IS assisted in study administration and data mining and UK planned and supervised the study, assisted in administration, data analysis and finalization of the manuscript. All authors read and approved the final version of the manuscript.
